# A doping-less junction-formation mechanism between n-silicon and an atomically thin boron layer

**DOI:** 10.1038/s41598-017-13100-0

**Published:** 2017-10-16

**Authors:** Vahid Mohammadi, Stoyan Nihtianov, Changming Fang

**Affiliations:** 1 Department of Microelectronics, Delft University of Technology, 2628 CD Delft, The Netherlands; 2 Brunel Centre for Advanced Solidification Technology (BCAST), Brunel University London, Uxbridge, Middlesex UB8 3PH UK

**Keywords:** Electronic devices, Sensors, Electronics, photonics and device physics

## Abstract

The interest in nanostructures of silicon and its dopants has significantly increased. We report the creation of an ultimately-shallow junction at the surface of n-type silicon with excellent electrical and optical characteristics made by depositing an atomically thin boron layer at a relatively low temperature where no doping of silicon is expected. The presented experimental results and simulations of the *ab initio* quantum mechanics molecular dynamics prove that the structure of this new type of junction differs from all other known rectifying junctions at this time. An analysis of the junction formation has led to the conclusion that the chemical interaction between the surface atoms of crystalline silicon and the first atomic layer of the as-deposited amorphous boron is the dominant factor leading to the formation of a depletion zone in the crystalline silicon which originates from the surface. The simulation results show a very strong electric field across the c-Si/a-B interface systems where the charge transfer occurs mainly from the interface Si atoms to the neighboring B atoms. This electric field appears to be responsible for the creation of a depletion zone in the n-silicon resulting in a rectifying junction-formation between the n-silicon and the atomically thin boron layer.

## Introduction

“Silicon, the backbone of modern electronics, is the most perfected, best understood and most heavily exploited electronic material of the information age we live in. The technological impact of new material systems with functional properties of relevance to semiconductor devices relies on their successful integration with silicon”^1^.

**Figure 1:**
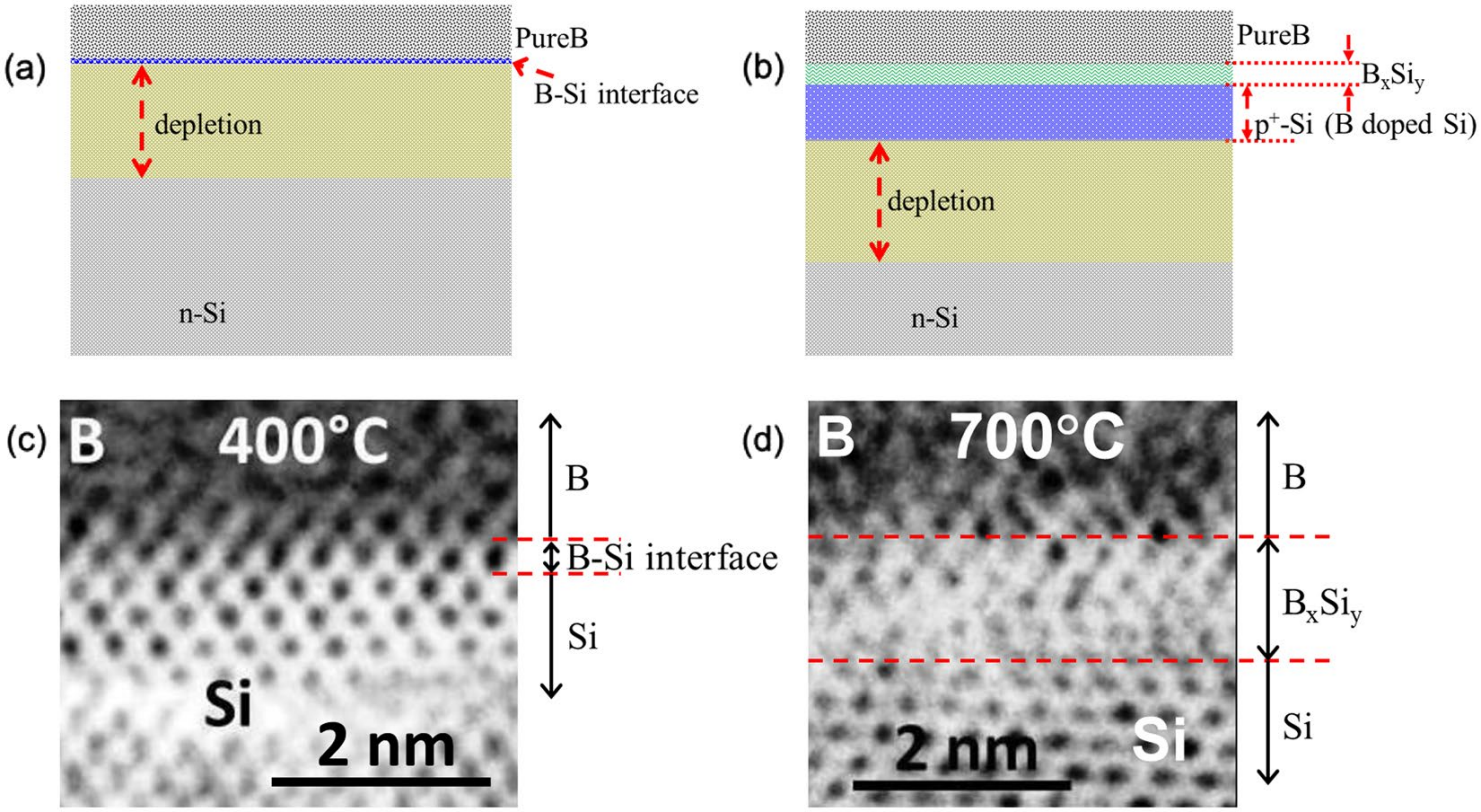
Schematic illustrations and HRTEM images of the cross-section of the layer stack. **(a**) Schematic illustration of the cross-section of the B-Si junction processed at 400 °C. (**b**) Schematic illustration of the cross-section of the B-Si junction processed at 700 °C. (**c**) HRTEM image of the cross-section of the B-Si junction processed at 400 °C. (**d**) HRTEM image of the cross-section of the B-Si junction processed at 700 °C.

It has been shown that a nanometer-thin boron amorphous layer can be created on the surface of crystalline silicon through a chemical vapor deposition (CVD) process in the temperature range from 700 °C to 400 °C^2^. At temperatures close to 700 °C, together with boron deposition, delta-doping of the silicon takes place^2^, as well as the formation of a 1–2 nm-thick layer of boron-silicide (B_x_Si_y_), leading to the formation of a shallow p-n junction. As in conventional p-n junctions, the depletion region is formed on either side of the junction, generating built-in potentials associated with uncompensated dopant atoms^3^. However, this temperature is too high for any existing CMOS structures, which makes the process CMOS-incompatible. By reducing the deposition temperature, the solid-solubility and diffusivity of the boron atoms in the silicon structure drop significantly^4,5^. At temperatures as low as 400 °C boron atoms no longer have sufficient kinetic energy to diffuse in crystalline silicon. Even if a small number of boron atoms manage to mix with the first few monolayers of silicon, this will still be insufficient to generate the number of holes needed to form a p-doped zone. This is logical given the high diffusion energy barriers (3.25 to 3.85 eV) and has been demonstrated both experimentally and in theoretical studies^6,7^. Diffusion originates from the gradient of the boron concentration in crystalline silicon. The diffusion coefficient *D* follows a relation resembling that of an exponential Arrhenius relation^6,7^:

1 $$D={{\rm{D}}}_{0}{e}^{(-{{\rm{E}}}_{{\rm{act}}}/{{\rm{k}}}_{{\rm{B}}}T)}$$

where E_act_ is the activation energy of the diffusion, D_0_ is the prefactor, k_B_ is the Boltzmann constant, and *T* is the absolute temperature in Kelvin. The high activation energy of the diffusion immobilises the boron at low temperatures, indicating strong temperature dependence, as shown in expression (1). For example, when the temperature drops from 700 °C (973 K) down to 400 °C (673 K) using E_act_ = 3.25 eV, the diffusion coefficient changes significantly: *D*_673K_/*D*_973K_ ~ 3.1 × 10^−8^ (more than seven orders of magnitude). Nonetheless, we have observed that the deposition of a nanometer-thin layer of amorphous boron on n-type silicon at 400 °C results in the formation of a rectifying B-Si junction due to a quantum mechanical phenomenon at the boron-silicon interface. The as-formed rectifying junction exhibits excellent electrical and optical characteristics^2^ without doping the silicon. Obviously, the structure of the B-Si junction differs from that of a p-n junction, as well as from all other currently known junctions. Figure 1 illustrates a schematic cross-section of the layer stack of the B-Si junction and the corresponding HRTEM images at deposition temperatures of 400 °C and 700 °C. Clearly, the HRTEM images confirm the formation of a 1–2 nm-thick boron-silicide (B_x_Si_y_) layer for the samples prepared at the high temperature (700 °C, Fig. 1d), while there is no such layer for the samples obtained using the low temperature process (400 °C, Fig. 1c).

Furthermore, a nanometer-thin boron layer can reliably protect the silicon, even in aggressive environments^8,9^. A very attractive feature of the junction-formation mechanism presented is its CMOS-compatibility, which makes it an excellent candidate for a wide range of applications, such as: low-penetration-depth radiation detection (low-energy electrons and vacuum-ultraviolet radiation)^2^; highly efficient solar energy harvesting^10^; high-speed linear varactors^11^; junction creation with wide-bandgap materials, e.g. SiC, without doping^2,12,13^; junction creation with promising new materials, e.g. graphene^14–17^.

## Results and Discussion

To support our hypothesis on the formation mechanism of the junction, understanding the interface structures and the local electronic properties between crystalline Si and the deposited B is of crucial importance. In this regard, theoretical approaches, especially the parameter-free first-principles methods, are very helpful and have been applied successfully for many different systems, including the interface systems between crystalline Si and both amorphous silicon oxide and nitrides^18–20^.

First-principles molecular dynamics (FP-MD) simulations based on the density functional theory (DFT) were performed for crystalline Si and amorphous boron (c-Si/a-B) interface systems. The simulations were performed in the following order.

First we heated the B layer at a high temperature (3000 K) in order to compensate for the short simulation time (~10 to 15 ps, pico-seconds). An analysis showed that the obtained B amorphous samples still exhibited both short-range and medium-range ordering, including distorted B_12_ icosahedra. This observation agrees well with previous simulations^21^.

**Figure 2:**
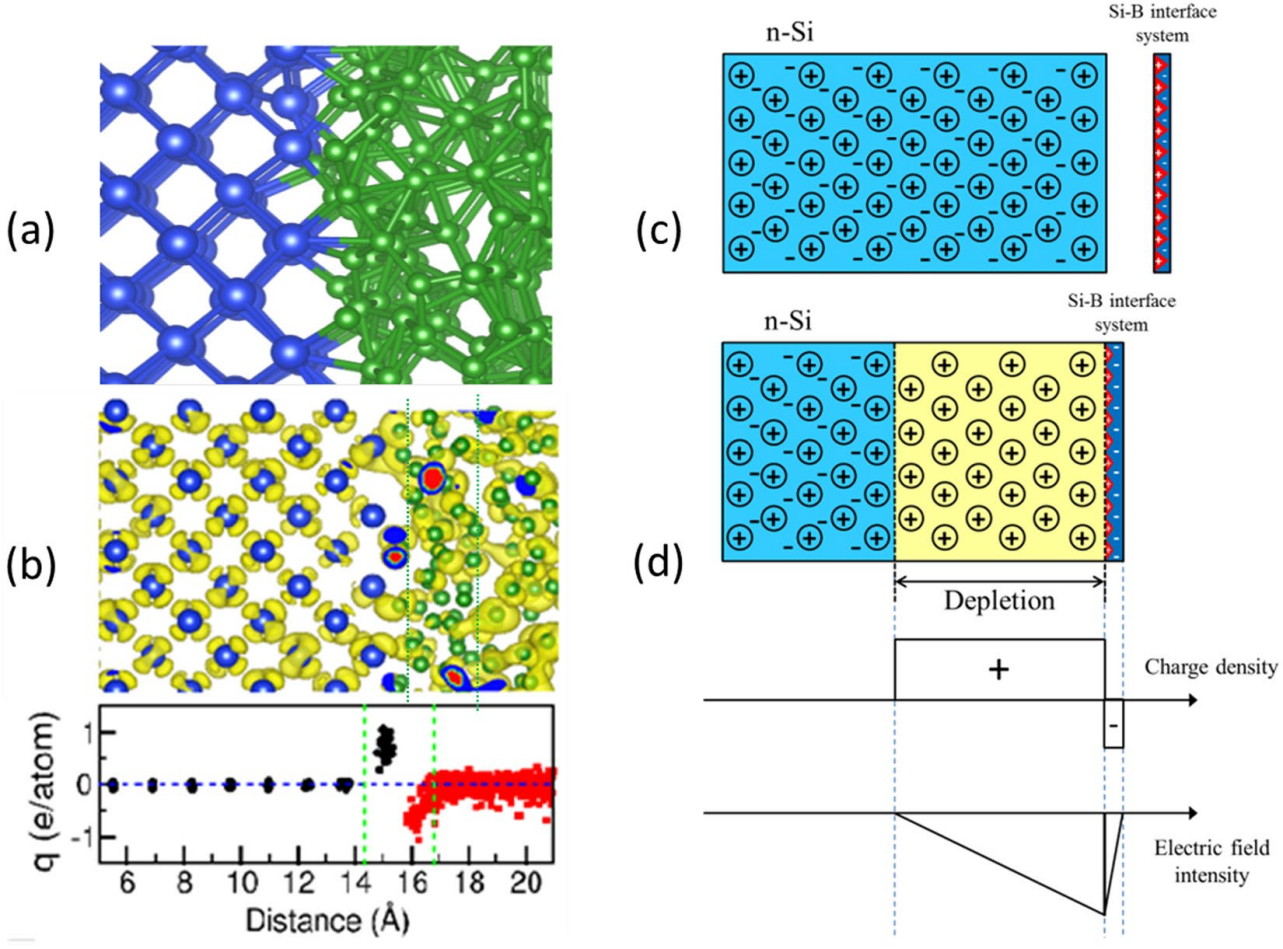
Formation of the B-Si junction. (**a**) Snapshot of the c-Si/a-B interface (blue spheres represent Si atoms, green spheres represent B atoms). (**b**) Density iso-surface for electrons near the Fermi level (top) and the charge distribution over Si (black dots) and B (red dots) atoms versus distance in angstrom (bottom). The blue horizontal line represents the neutral value (bottom) and the green lines represent the charge range. (**c**) n-Si and Si-B interface system before interaction. (**d**) Formation of the depletion zone due to charge redistribution between the bulk n-Si and the 2D Si-B interface, after interaction; charge density; electric field intensity.

Next, the c-Si/a-B systems created were gradually cooled down to 0 K in about 10 to 20 ps. Finally the systems were relaxed to eliminate the internal forces. A structure analysis showed that, at the interfaces, various chemical bonds and an interface B atom may be connected to one or two Si atoms beyond the B-B bonds (see Fig. 2a; blue spheres represent Si atoms, green spheres represent B atoms). Therefore, the interfacial B has three to five neighbours, similar to both those in the amorphous bulk and those in the crystalline B phases. This chemically strong Si-B bonding indicates a strong mechanical Si-B interface. This agrees with the experimental observations.

The B-Si junction formation can be described with the charge transfer which occurs at the c-Si/a-B interface. The notable difference in electronegativity (2.04 for B and 1.90 for Si) indicates ionicity of the Si-B bonds. Therefore we employed the Bader charge analysis approach^22–24^. Our simulations show that for c-Si/a-B interface systems, the charge transfers mainly from the interface Si atoms to the neighbouring B (Fig. 2b). Statistics show that lost electron values of the interface Si ions range from 0.40 to 0.97 e/Si with an average value of 0.76 e/Si. For un-doped or p-type c-Si/a-B interfaces, the charge transfer is limited to the interfaces and thus no rectifying effects occur due to the small width of the interface regions (~0.2 nm) as compared to the free path of electrons (larger than 1 nm for electrons 1 to 10 eV in energy)^25^.

When a few monolayers of a-B are deposited on n-type c-Si (n-Si), diffusion of free electrons takes place from the n-zone to the localized two-dimensional (2D) p-zone due to Coulomb interactions (Fig. 2c and d). The electric field at a distance r (r ≫ d) decays with distance from the Si-B interface, similar to the electric field outside a parallel plate capacitor^26^. When an electron from the n-Si moves to the interface, a static uncompensated positive charge appears locally. This positive charge balances the attraction from the interface and in this way a static balance is reached.

To obtain more insight into the nature of the B-Si junction and its electrical and optical properties, structures with a large active area (9 mm × 9 mm) were processed in two separate runs using a CVD process to deposit amorphous boron on n-type silicon (doping level 1 × 10^16^/cm^3^), in two different reactors. The nanometer-thin boron was used as a capping and protective layer, and as a window for the optical characterization tests. The electrical contact with the boron layer was realized with a “ring” electrode – a narrow metalized strip positioned at the edge of the photo-diode active area.

The measured I-V characteristics of 50 B-Si photodiodes prove the excellent electrical properties of this rectifying junction^2^. The average measured saturation current density (with a reverse bias voltage of −1 V) was 0.6 pA/mm^2^. The breakdown voltage measured was higher than 30 V. We also performed C-V measurements of the junction capacitance as a function of the bias voltage. The measured capacitance without the bias voltage was ~1 nF, from which we calculated a depletion zone width of ~8 μm. From these data we can roughly predict the amount of charge in the depletion region: q_dep_ = 1 × 10^16^ e/cm^3^ × 8 μm = 8 × 10^12^ e/cm^2^ if we assume a complete depletion, or q_dep_ = 4 × 10^12^ e/cm^2^ if we assume a linear decay model. The Si density at the Si surfaces is about 7 × 10^14^ atoms/cm^2^. At this point the change in charge at the interface can be calculated: (8 × 10^12^ e/cm^2^)/(7 × 10^14^ atoms/cm^2^) ~ 0.01 e/Si for a complete depletion, or 0.005 e/Si for a linear decay model. Even though the results presented do not imply very high precision due to the uncertainty in the depletion width estimation, we can conclude that the static balance is achieved with an insignificant change in charge in the B-Si bonding, and therefore has little impact on the chemical and physical properties.

In the B-Si junction, the role of the p-doped zone is performed by the B-Si monolayer. For applications such as photo-detection, the photo-generated charge is removed from the photodiode by the ring electrode. To reach the ring electrode, the photo-generated charge in the central zone of the photodiode active area must follow a horizontal trajectory after being separated by the electric field in the depletion zone. This happens mostly in the B-Si monolayer. Therefore it is important to know the mobility of the charge passing through the B-Si monolayer and the related sheet resistance. Our electronic band structure calculations show that even at the region closest to the B-Si interface, the mobility of the electrons is still quite high, and their effective mass component perpendicular to the interface is roughly two or three times that of the free electron. The measured sheet resistance is ~60 kΩ/square, which is expectedly higher than that of photodiodes with a p^++^ top layer^2^.

**Figure 3:**
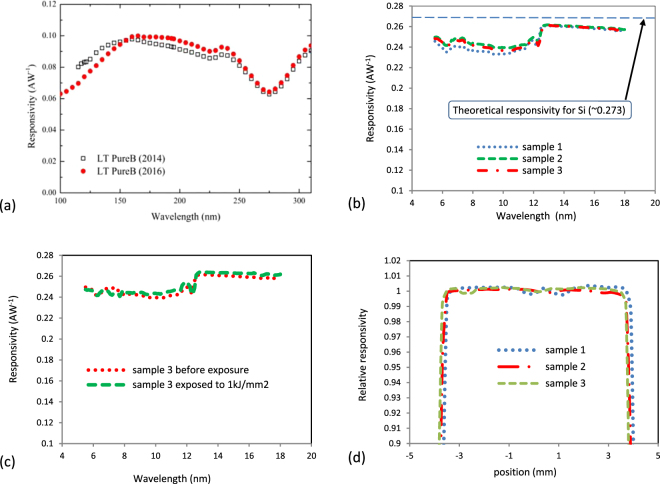
Optical characteristics of the B-Si junction. (**a**) Measured spectral responsivity in the near-ultraviolet and vacuum-ultraviolet spectra. The responsivity of the same B-Si photodiode was measured over two years to confirm the stability of the B-Si junction over time. **(b**) Measured spectral responsivity of three samples from one wafer in the extreme ultraviolet spectrum. (**c**) Measured responsivity of Sample 3 before and after exposure to 1 kJ/mm^2^. (**d)** Typical spatial uniformity of responsivity, measured along the horizontal axis of the samples at 13.5 nm wavelength irradiation.

The spectral responsivity and stability of B-Si photodiodes were measured not only in the challenging near-ultraviolet and vacuum-ultraviolet spectrum ranges where the penetration depth of photons in silicon and boron is only a few nanometers, but also in the extreme ultraviolet spectrum range where the photons have strong ionizing properties (Fig. 3). Figure 3a shows the responsivity of a B-Si photodiode measured twice over two years (2014 and 2016) to confirm the stability of the B-Si junction over time. The small variation between the two curves is mainly due to the measurement uncertainty. Figure 3c shows the measured responsivity of Sample 3 before and after undergoing a significant exposure to 1 kJ/mm^2^. The small difference between the two spectral responses is within the uncertainty of the measurement, which demonstrates exceptional radiation hardness. Figure 3d shows the spatial uniformity of responsivity of the three samples. The scan was done along the horizontal axis of the samples with 13.5 nm irradiation. The measured non-uniformity was within ±0.5%, which is very close to the uncertainty of the measurement tool.

From the reported electrical and optical performance it is evident that the B-Si diodes have excellent electrical characteristics and demonstrate very high sensitivity and stability in the UV spectrum. The clean-room equipment and materials used, as well as the low boron deposition temperature, make this process CMOS-compatible. Being one of the last processing steps, it will not harm the CMOS structures already built on the same wafer.

## Methods

### DFT computational approach

A supercell of the Si(001)/a-B system is composed of 12 layers of Si atoms separated by a thin layer of a-B. Each layer contains 8 Si atoms in a supercell of 2*a*_0_ × 2*a*_0_ × 8.34*a*_0_; *a*_0_ is the lattice parameter of Si. The density of B is the same as that of tetrahedral β-B_105_. Therefore, this cell contains 96 Si and 100 B atoms. The system was built with the following two steps.

Step one involved placing 100 B atoms in a cell with a density identical to that of tetrahedral β-B_105_ (2*a*_0_ × 2*a*_0_ × 1.34*a*_0_). The B was then heated to 4000 K (melting point for B is 2349 K) and kept at that temperature for 6,000 steps (6 ps). Next, the sample was slowly cooled to 3000 K in 8,000 steps (8 ps). Then the obtained a-B was inserted into a Si slab. The assembled Si(001)/a-B system was heated at 3000 K again and kept at that temperature for 14,000 iterations (14 ps) with the Si part fixed. We took several samples from the last 3 ps at intervals of about 300 iterations or 300 fs. Those samples were cooled from 3000 K to room temperature in 3 to 10 ps. Finally, the samples were relaxed to eliminate the internal forces.

For step two, the first-principles code VASP (Vienna Ab initio Simulation Program)^27,28^ was employed for all the calculations. This code uses periodic boundary conditions (PBC). The density functional theory (DFT) was used with the Projector Augmented Wave (PAW) method and the Generalized Gradient Approximation (GGA)^29^ digitized by Perdew, Burke and Ernzerhoof (PBE). The cut-off energy of the wave functions was 500 eV, while the cut-off energy of the augmentation functions was 700 eV. The electronic wave functions were sampled on a 4 × 4 × 2 grid with 20 k-points in the irreducible Brillouin zone (BZ) of the supercell for structural relaxation and electronic properties calculations, including a Bader charge analysis, using the Monkhorst–Pack method^30^. A Γ-only k-mesh was employed for molecular-dynamics simulations. The cut-off energy of the wave functions was reduced to 250.0 eV. The time interval was 1 femtosecond (fs) for each step.

### Boron deposition on silicon

**Figure 4:**
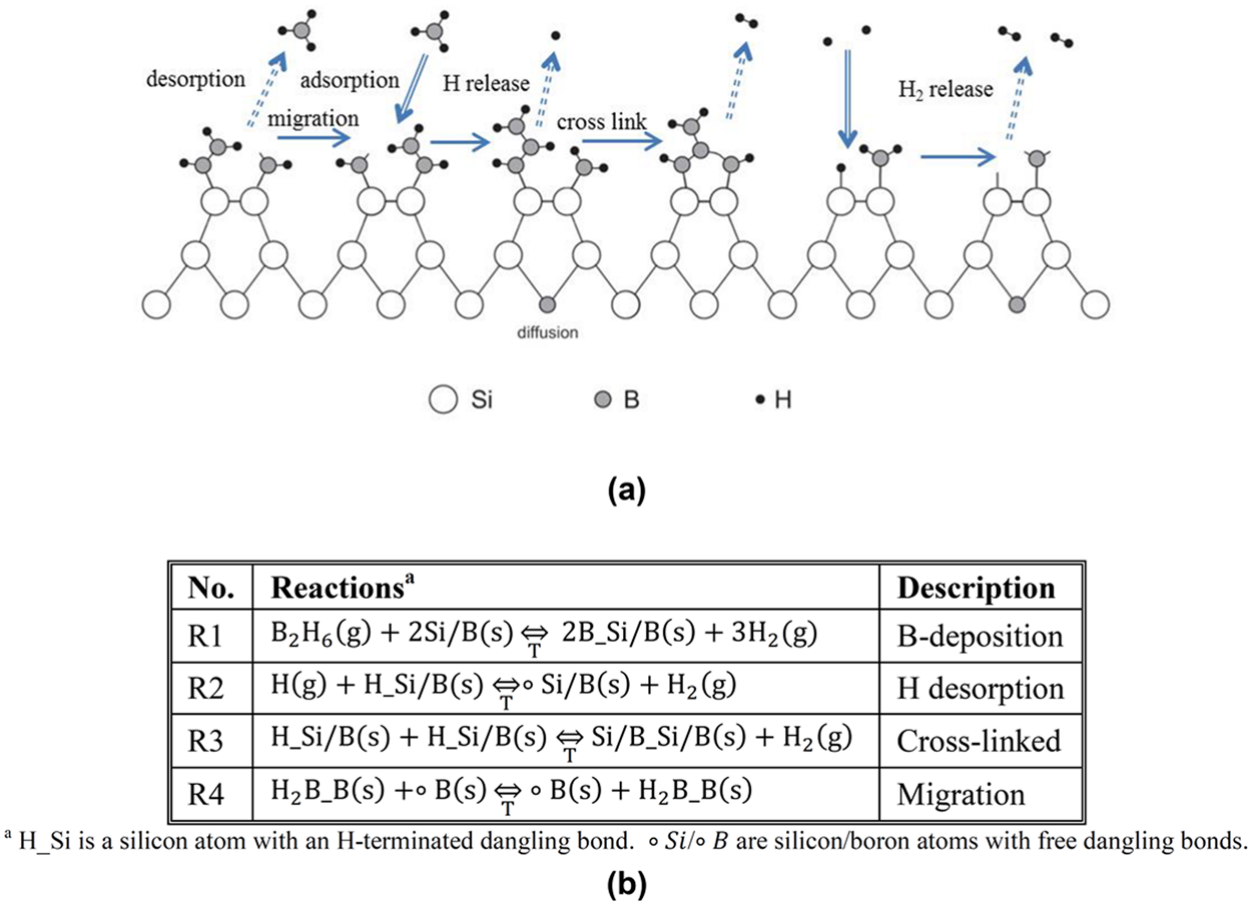
Chemical reactions during CVD boron deposition. (**a**) Schematic. (**b**) Table with reactions.

For the formation of the B-Si junction, some *ex-situ* and *in-situ* processing steps are necessary. The *ex-situ* steps involve removing oxides and contaminants at the Si surface and effectively passivating the surface^2^. Prior to depositing the boron over the prepared clean Si surface, to form the B-Si junction, some other processing steps are necessary as *in-situ* steps^2^. First the H must be desorbed from the Si surface to leave H-free surface Si sites with dangling bonds, and then certain reactions must take place to deposit the boron atoms over these Si sites as shown schematically in Fig. 4a and summarized in the Table in Fig. 4b^31^.

### Processing data of the sample used in Figure 3a

The sample used in Fig. 3a was fabricated using a post-metal 400 °C boron CVD process, called the LT (low-temperature) PureB process. The deposition was performed in a commercial ASMI Epsilon 2000 Si/SiGe epitaxial reactor using diborane as the precursor and a combination of H_2_ and N_2_ carrier gases designed to maximize the deposition rate and minimize layer roughness.

**Figure 5:**
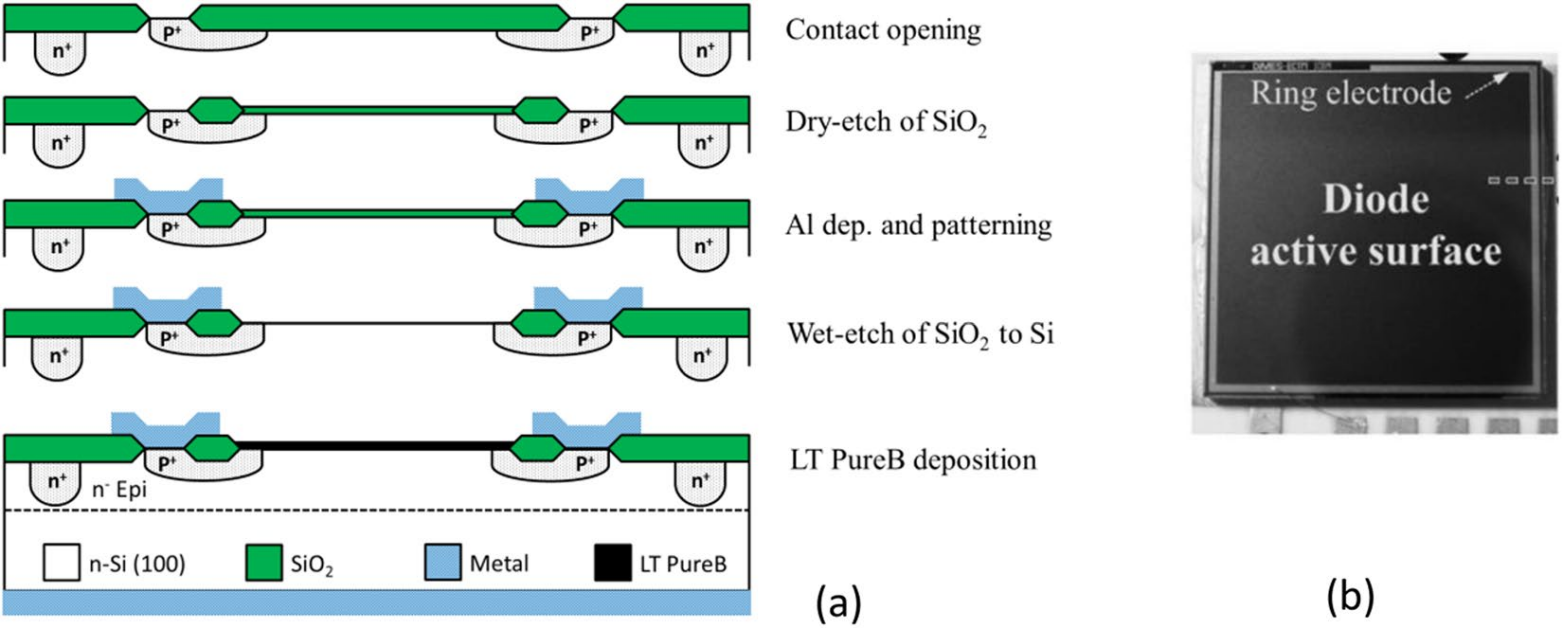
Photodiodes used in Fig. 3a. (**a**) Processing steps of the fabricated photodiodes with boron-only beam entrance windows, for boron deposition after the metallization. (**b**) Photograph of the photodiode with a 9.6 mm × 9.6 mm active area.

In Fig. 5 the step sequence of the basic process flow is given for the fabrication of the photodiode. The boron was deposited after metallization, thanks to the low deposition temperature. In this case the contact with the boron layer was made via a p^+^ guard ring which was implanted and annealed before metallization. This process flow also had the advantage that the as-deposited boron layer directly formed the beam-entrance window and did not require any post-processing, such as metal removal, which is a challenging step.

### Processing data of the samples used in Figures 3b and c

**Figure 6:**
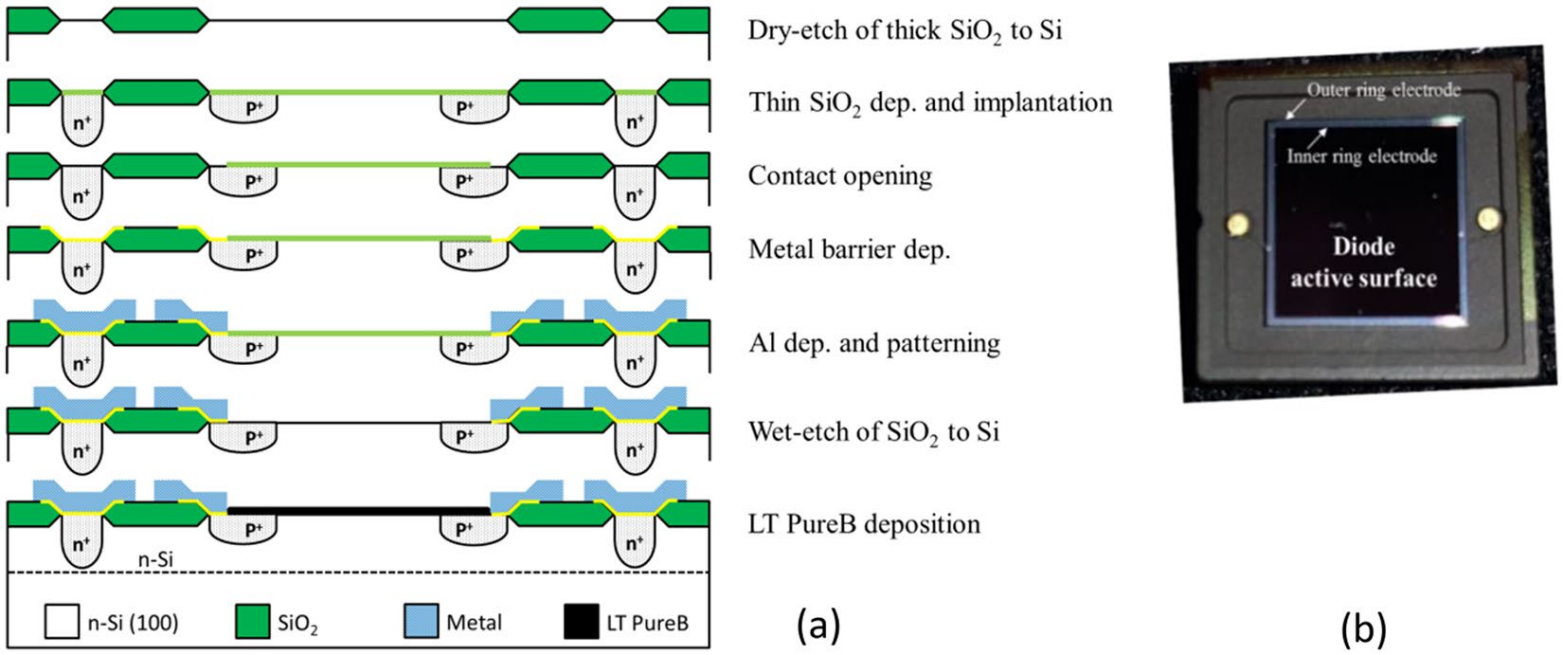
Photodiodes used in Fig. 3b. (**a**) Schematic of the fabricated photodiodes with PureB-only beam entrance windows for PureB deposition after the metallization. (**b**) Photograph of the photodiode in a Kyocera ceramic package.

The samples used in Fig. 3b were also fabricated using the post-metal LT PureB process. This time the boron deposition was performed in a commercial PECVD Applied Materials Centura DXZ 200 mm reactor. Figure 6a shows the processing steps for fabricating the photodiodes, which were part of a standard CMOS process. These samples were designed to have both the anode and cathode contacts on the front side. Figure 6b shows a photograph of a photodiode in a Kyocera ceramic package.

### C-V measurements

**Figure 7:**
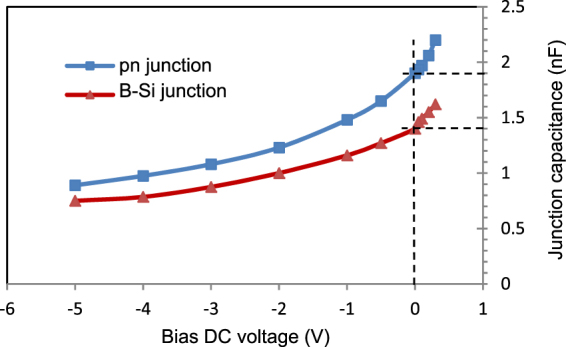
Typical C-V characteristics of a high-temperature (HT) p-n junction and a low-temperature (LT) B-Si junction. The two junctions have the same layout, but are processed in separate wafer batches. The two C-V characteristics have similar shapes. The difference in the junction capacitance values, which defines the different depletion widths, is most likely due to variations in the doping levels of the n-type silicon of the two wafer batches.

To define the depletion width of the B-Si diode, C-V measurements were performed. Similar C-V measurements were performed using diodes with the same layout, but processed with the high-temperature (HT) PureB CVD process resulting in a p-n junction. The measurement results are presented in Fig. 7. The C-V characteristics were measured with the Impedance Analyser HP4294A. The measured impedance was modelled as a capacitor C_s_ in series with a resistor R_s_. The capacitor and the resistor represent the junction capacitance and the series resistance of the diode, respectively. An AC signal with a frequency of 2 MHz was used with fixed amplitude of 10 mV and superimposed on a DC bias voltage with different values. To calculate the depletion width of the B-Si diode, we used a parallel-plate capacitance model with the following parameters: electric constant of ε_0_ = 8.854 × 10^−12^ F, relative permittivity of ε_r_(Si) = 11.7, and diode area of 81 mm^2^.

### I-V measurements

**Figure 8:**
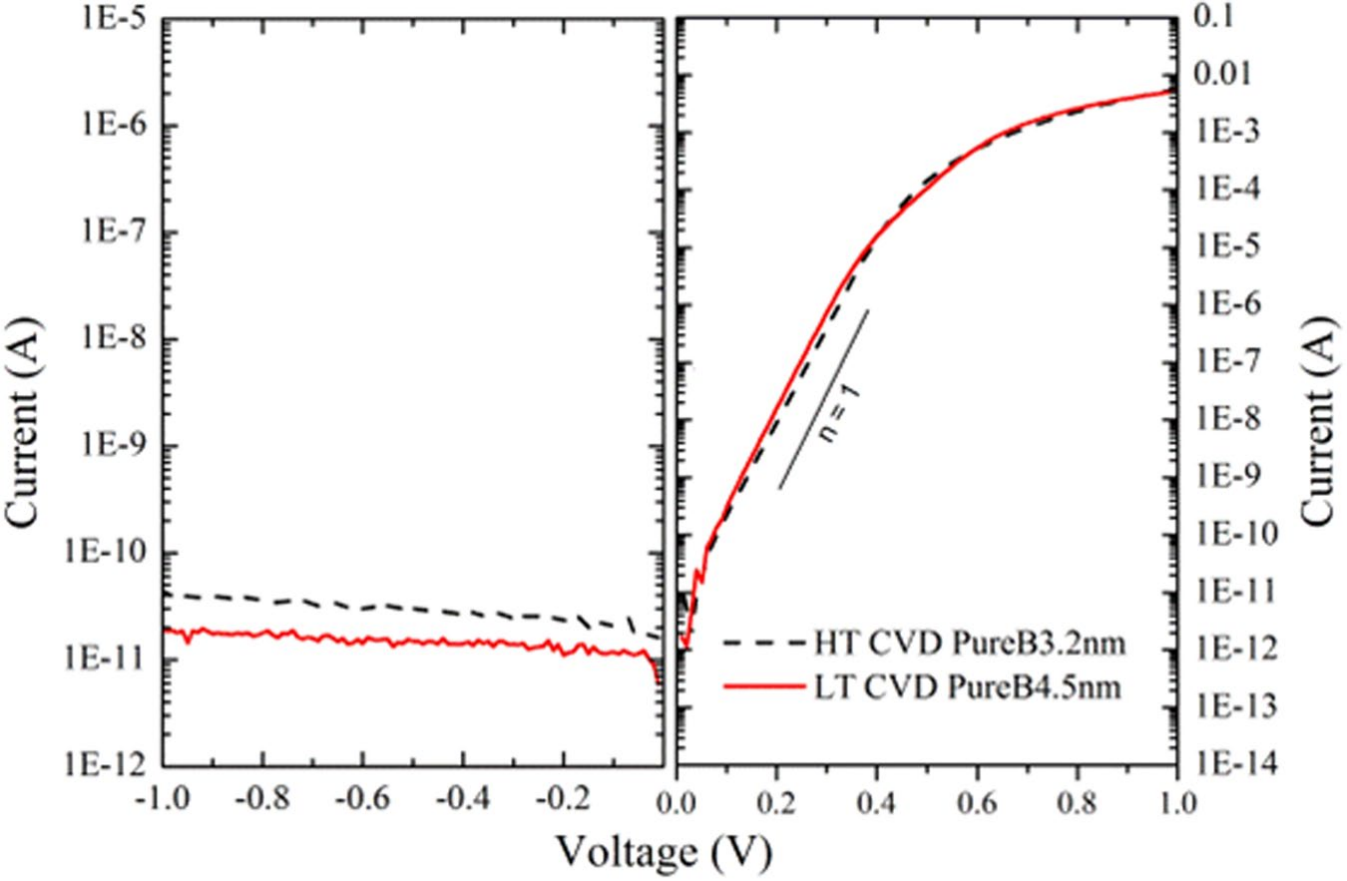
Measured I-V characteristics of the B-Si diode samples. The I-V measurements of pre-metal HT- and post-metal LT-PureB photodiodes with an active area of (9.6 × 9.6) mm^2^. The boron layer thickness was measured by ellipsometry and found to be 3.2 nm and 4.5 nm for the HT and LT photodiodes, respectively.

The measured I-V characteristics of both pre-metal HT (700 °C) and post-metal LT (400 °C) PureB photodiodes with an active area of (9.6 × 9.6) mm^2^ are shown in Fig. 8. The boron layer thickness was measured by ellipsometry and found to be 3.2 nm and 4.5 nm for the HT and LT photodiodes, respectively. As can be seen, low deep-junction-like saturation currents and near-ideal diode characteristics can be provided by LT boron deposition.

### Spectral responsivity measurements

The spectral responsivity results presented in Fig. 3 were obtained at the National Metrology Institute of Germany - PTB (Physikalisch-Technische Bundesanstalt). Synchrotron radiation in the UV and VUV spectra was used from the electron storage ring Metrology Light Source (MLS) and synchrotron radiation in the EUV spectrum was used from the electron storage ring BESSY II, both located in Berlin-Adlershof,

### Electronic band structure

**Figure 9:**
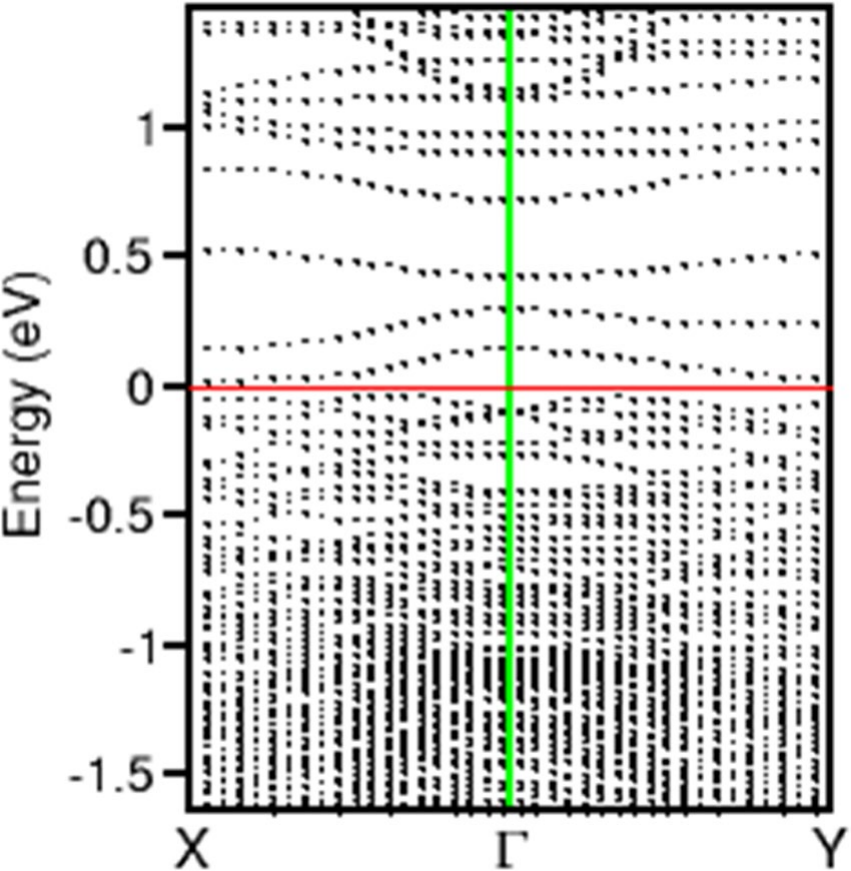
Electronic band structure. Dispersion curves along the in-plane orientations for one Si(001)/a-B configuration along the line X-Γ-Y in the Brillouin zone. The Fermi level (the red horizontal line) is set at zero eV. The vertical green line represents the electronic states at Γ.

**Figure 10:**
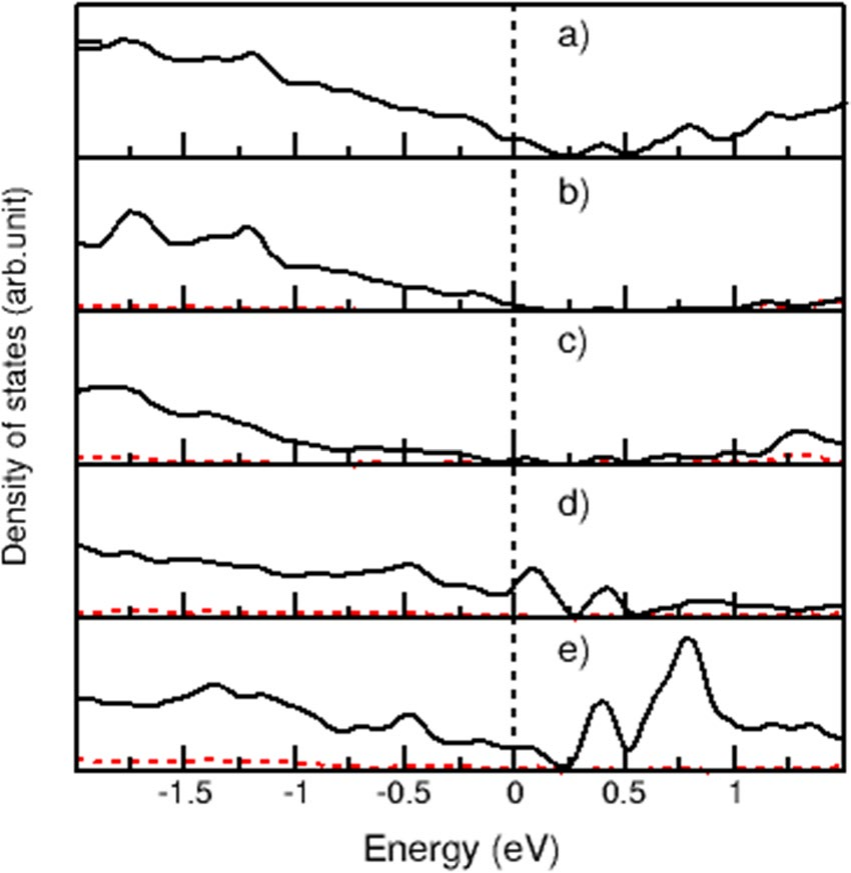
Electronic band structure for the a-B/c_Si system with the dotted red /solid black lines represent the 2 s/2p- characters for B and Si atoms/ions: (**a**) Total densities of states for a bulk-line Si. (**b**) Partial densities of states for a bulk-line Si. (**c**) Partial density of states for one boron in amorphous form. (**d**) Partial density of states for a Si at the c-Si/a_B interfac. (**e**) Partial density of one B at the c-Si/a_B interface.

Electronic band structure calculations were also performed for the c-Si/a-B interface system. A typical electronic structure of the c-Si/a-B interface interface is shown in Fig. 9 (dispersion curves) and 10 (density of states). Figure 10 compares the partial density of states (pDOS) for a Si (or B) atom at the interface with one in the corresponding centre. There are notable differences for the pDOS of the Si at the interface (Fig. 10d) and that in the centre (Fig. 10b). The pDOS of the amorphous B atom the interface (Fig. 10e) is also notably different from that of the B in amorphous (Fig. 10c). Clearly there are several defect states at the forbidden gap of the Si. The partial density of states (pDOS) for the Si at the interface two small peak at about 0.1 and 0.4 eV which are the unoccupied Si 2p states. The one at 0.1 eV corresponds to the lowest unoccupied state which is just above the Fermi level at the X point (Fig. 9). This result agrees with the eigen-character analysis that this state belongs to the interface Si atoms (Fig. 10d) with some contribution from the related B atoms. Therefore, it is concluded that charge transfer occurs from the interface Si to related B, which is crucial for forming Si-B junctions.
