# Author Correction: A doping-less junction-formation mechanism between n-silicon and an atomically thin boron layer

**DOI:** 10.1038/s41598-021-99821-9

**Published:** 2021-10-12

**Authors:** Vahid Mohammadi, Stoyan Nihtianov, Changming Fang

**Affiliations:** 1 Department of Microelectronics, Delft University of Technology, 2628 CD Delft, The Netherlands; 2 Brunel Centre for Advanced Solidification Technology (BCAST), Brunel University London, Uxbridge, Middlesex UB8 3PH UK

Correction to: *Scientific Reports* 10.1038/s41598-017-13100-0, published online 16 October 2017

Whilst the original version of this Article cited Mohammadi’s related thesis as reference 2, the relevant original literature covering the technological aspects were not. As a result, references 32 – 35 are omitted and are listed below.

32. Mok, K.R.C., Mohammadi, V., Nanver, L.K., de Boer, W.D., & Vlooswijk, A.H.G. Low-pressure chemical vapor deposition of PureB layers on silicon for p+n junction formation. In *12th International Workshop on Junction Technology, Shanghai, China*, 113-116 https://doi.org/10.1109/IWJT.2012.6212822, 113-116 (2012).
33. Nanver, L.K. *et al.* Pure dopant deposition of B and Ga for ultra-shallow junctions in Si-based devices. *ECS Trans*. **49**, 25 (2012).
34. Mohammadi, V. *et al.* VUV/low-energy-electron Si photodiodes with post-metal 400 °C PureB deposition. *IEEE Electron. Device Lett*. **34**, 1545 (2013) DOI:https://doi.org/10.1109/LED.2013.2287221 (2013).
35. Nanver, L.K. *et al*. Robust UV/VUV/EUV PureB photodiode detector technology with high CMOS compatibility. *IEEE J. Sel. Top. Quantum Electron*. **20**, 306-316. DOI:https://doi.org/10.1109/JSTQE.2014.2319582i (2014).

In addition, reference 36, which discusses an alternative junction formation mechanism, was omitted and is listed below.

36. Qi, L. and Nanver, L.K. Conductance along the interface formed by 400 °C pure boron deposition on silicon. *IEEE Electron. Device Lett.* **36**, 15102. DOI:https://doi.org/10.1109/LED.2014.2386296 (2015).

Consequently, the sentence in the Introduction,

“It has been shown that a nanometer-thin boron amorphous layer can be created on the surface of crystalline silicon through a chemical vapor deposition (CVD) process in the temperature range from 700 °C to 400 °C^2^.”

should read:

“It has been shown that a nanometer-thin boron amorphous layer can be created on the surface of crystalline silicon through a chemical vapor deposition (CVD) process in the temperature range from 700 °C to 400 °C^2,32–36^.”

And the text,

“The as-formed rectifying junction exhibits excellent electrical and optical characteristics^2^ without doping the silicon.”

should read:

“The as-formed rectifying junction exhibits excellent electrical and optical characteristics^2,36^ without doping the silicon.”

Finally, in the Methods section, under the subheading “Boron deposition on silicon”, the sentence

“For the formation of the B-Si junction, some *ex-situ* and *in-situ* processing steps are necessary. The *ex-situ* steps involve removing oxides and contaminants at the Si surface and effectively passivating the surface ^2^.”

should read:

“For the formation of the B-Si junction, some *ex-situ* and *in-situ* processing steps are necessary. The *ex-situ* steps involve removing oxides and contaminants at the Si surface and effectively passivating the surface^2,32–35^.”
